# Editorial: Insights in heart failure and transplantation: 2022

**DOI:** 10.3389/fcvm.2023.1290011

**Published:** 2023-09-21

**Authors:** Matteo Cameli, Emma J. Birks, Federico Landra

**Affiliations:** ^1^ Division of Cardiology, Department of Medical Biotechnologies, University of Siena, Siena, Italy; ^2^ Division of Cardiovascular Medicine, University of Kentucky, Lexington, KY, United States

**Keywords:** heart failure, heart transplant, cardiac surgery, donation, diabetes

**Editorial on the Research Topic** Insights in heart failure and transplantation: 2022

## Heart transplant: expanding the donor pool

Just over 50 years from the first Heart Transplant ever performed by Dr Christiaan Barnard in 1967 in Cape Town, heart transplantation has become the mainstay therapy for patients with advanced heart failure. Today, the main limitation of the applicability of such treatment is the well-recognized shortage of organ donors in the modern era. Not only are donor hearts lacking but also the number of patients requiring a heart transplant is incessantly increasing, due to population aging and improved survival of patients living with heart failure. As a result, between 5% and 10% of patients die while on waiting lists.

Even though bridge solutions to heart transplant are becoming more and more familiar with left ventricular assist devices, the problem overall remains largely unsolved. Different proposals have been advanced over the last decades trying to face this brain teaser. Some of them have already become reality in some countries, such as acceptance of hearts from HCV-positive donors, thanks to curative treatments now available, and protocols to widen the spectrum of donors. Particularly, the ADONHERS protocol, developed in Italy in Emilia-Romagna and Tuscany regions, aims at assessing the eligibility of the so-called marginal donors, namely those with >55 years or <55 years with multiple cardiovascular risk factors, employing stress-echocardiography to rule out subtle coronary artery disease (Cameli et al.).

Other promising solutions not widely used yet are *ex-vivo* heart perfusion platforms and donation after circulatory death, the latter coming as a revolutionary paradigm. Actually, the first heart transplant performed by Dr Barnard was from a donation after circulatory death and at the beginning of heart transplant history donation after circulatory death was common practice. Later, with the introduction of brain-death legislation, donation after brain death became the standard method, which also permitted to minimize organ hypoxia. Nowadays, heart transplant is routinely performed from brain-dead donors using cold storage, but from the early 2000 donation after circulatory death has raised renewed interest following successful experience from abdominal and pulmonary transplantations. Donation after circulatory death is performed in patients who do not fulfill brain death criteria but have no chance for recovery. The main difference with donation after brain death is the occurrence of warm ischemia after withdrawal of life support. Nowadays, substantial body of research has been done to limit the ischemic injury by different protocols. Recent clinical data suggest noninferiority compared to donations after brain death, making donation after circulatory death a potential solution to the shortage of organs.

## Cardiac surgery after heart transplantation

The largest available dataset of heart transplant patients undergoing cardiac surgery from three different continents and sixty high-volume centers has been published in this Research Topic (Gökler et al.). One hundred ten patients have been collected and results show valvular disease to be the most common indication for cardiac surgery in this special population. Among them, tricuspid valve disease was the one most largely observed, mostly as a results of intense surveillance protocols requiring frequent endomyocardial biopsies to rule out rejection. Another relatively common indication was coronary artery vasculopathy, even though percutaneous coronary intervention is usually preferred in this case. Surgery in heart transplant patients may be challenging because of surgical reintervention and may be complicated by a higher rate of infections due to the immunosuppressive regimens. For these reasons, surgery after heart transplantation is rarely performed unless in highly selected cases. According to data from this register, the Authors conclude that surgery in this context is relatedly safe, with low in-hospital mortality and postoperative complications in carefully selected patients. Nonetheless, the overall in-hospital and 1-year mortality after surgery were 9.1% and 13.8%, respectively, which are not neglectable after all. Therefore, the surgical option is certainly feasible but not free from safety concerns and should be considered only in specific conditions with indubitable benefit as compared to the interventional alternative.

## Diabetes in heart failure with preserved ejection fraction

The relevance of comorbidities in patients with heart failure with preserved ejection fraction is already largely recognized. Treatment of non-cardiovascular comorbidities has recently received a class I recommendation in the latest ESC Guidelines for the diagnosis and treatment of acute and chronic heart failure for patients with a preserved ejection fraction. Diabetes is one of the major risk factors for cardiovascular diseases and specifically for heart failure. There is a close interplay between diabetes and heart failure which is not completely understood yet. Complex pathophysiological processes may eventually lead to heart failure in diabetic patients, also independently from the presence of ischemic heart disease or hypertension, which has led to the discussed definition of diabetic cardiomyopathy in the past years. Beside the hermetic etiological process, heart failure patients with concomitant type 2 diabetes experience a more relevant reduction in the functional capacity. Also, diabetes showed to be the most powerful predictor of limited exercise capacity in patients with heart failure with preserved ejection fraction (Berisha-Muharremi et al.).

Sodium-glucose co-transporter 2 inhibitors were originally thought to be used in diabetic patients, but they have unexpectedly seen a massive spread among the cardiological community because of their clear benefit in patients with heart failure. Initially their use has been assessed in patients with a reduced ejection fraction, but recent randomized controlled trails have shown significant prognostic benefit also for that orphan disease which is heart failure with preserved ejection fraction. Indeed, besides diuretics for fluid retention, no drugs have ever proved benefit in this subset of patients. Sodium-glucose co-transporter 2 inhibitors come as the first specific therapy, notably with a class I recommendation, for patients with a preserved ejection fraction. As such, they represent the only drugs with a class IA recommendation across the whole range of ejection fraction in patients with heart failure. A systematic review and meta-analysis from Treewaree et al. published in the present issue has proved their benefit in terms of improvement of cardiovascular outcomes and quality of life in patients with heart failure with preserved and mildly reduced ejection fraction, anticipating the proposed recommendations in the 2023 Focus Update of the 2021 ESC Guidelines for the diagnosis and treatment of acute and chronic heart failure.

## Final considerations

Much evidence regarding both heart transplant and heart failure is continuously emerging, providing deeper insights into diseases' pathophysiology which eventually improve their clinical management. Aside from the papers highlighted herein, many other high-quality works have been published in this topic which well deserve a lecture. From biomarkers to echocardiography, from cardiac resynchronization therapy to left ventricular assist devices, this Research Topic covers a wide range of important subjects concerning heart failure.

